# Effects of SGLT2 Inhibitors on Proteinuria and Renal Function Parameters in Non-Diabetic Kidney Transplant Recipients: A Retrospective Cohort Study Based on 12-Month Follow-Up Data

**DOI:** 10.3390/jcm15135303

**Published:** 2026-07-07

**Authors:** Serdar Kahvecioglu, Huseyin Celik, Asena Serap Karatutlu, Saide Elif Gullulu Boz, Pinar Ozdemir, Ozger Akarsu, Nazife Nur Ozer Sensoy, Nimet Aktas

**Affiliations:** 1 Department of Nephrology, Bursa Yüksek İhtisas Training and Research Hospital, 16260 Bursa, Türkiyeelifgullulu@uludag.edu.tr (S.E.G.B.);; 2 Department of Nephrology, Bursa Acıbadem Hospital, 16210 Bursa, Türkiye; 3 Department of Internal Medicine, Bursa Yüksek İhtisas Training and Research Hospital, 16260 Bursa, Türkiye

**Keywords:** kidney transplantation, non-diabetic, proteinuria, renal function, SGLT2 inhibitors

## Abstract

**Background**: Sodium–glucose cotransporter 2 (SGLT2) inhibitors have demonstrated significant renoprotective effects in patients with chronic kidney disease. However, kidney transplant recipients have been excluded from major randomized trials, and evidence in non-diabetic transplant patients remains limited. This study aimed to investigate the potential effects of SGLT2 inhibitors in non-diabetic kidney transplant recipients. **Methods**: Kidney transplant recipients were screened retrospectively and divided into two groups based on SGLT2 inhibitor use. A total of 18 non-diabetic patients receiving SGLT2 inhibitors (Group 1) were compared with 30 matched controls (Group 2). Patients were followed at baseline, 3, 6, and 12 months. Proteinuria, serum creatinine, eGFR, uric acid, and tacrolimus levels were analyzed. **Results**: Baseline demographic and biochemical characteristics were similar between groups. In Group 1, proteinuria decreased by 20% at 6 months and 26% at 12 months compared with baseline. The reduction in proteinuria from baseline to 6 months was significantly greater in Group 1 than in controls (*p* = 0.037). No significant changes were observed in serum creatinine, eGFR, tacrolimus levels, or infection-related adverse events between groups. **Conclusions**: SGLT2 inhibitors may confer an early antiproteinuric benefit in non-diabetic kidney transplant recipients without apparent adverse effects on renal function or safety. Larger prospective studies are needed to confirm long-term effects.

## 1. Introduction

Kidney transplantation is the preferred treatment modality among renal replacement therapies [1]. Although post-transplant outcomes are generally favorable, graft function may deteriorate due to both early and late complications. Multiple factors, including ischemia–reperfusion injury, oxidative stress, drug-related toxicity, and recurrence of the primary disease, may contribute to this deterioration [2]. Such deterioration is commonly reflected by increasing serum creatinine levels and the development of proteinuria. Despite advances in transplant care, there is still no definitive therapeutic strategy to halt the progression of chronic changes detected on biopsy.

In recent years, substantial benefits have been demonstrated in non-transplant chronic kidney disease (CKD) populations with sodium–glucose co-transporter 2 inhibitors (SGLT2is), glucagon-like peptide-1 (GLP-1) receptor agonists, and next-generation mineralocorticoid receptor antagonists (MRAs), either alone or in combination with angiotensin-converting enzyme inhibitors (ACEis) or angiotensin II receptor blockers (ARBs) [3,4,5,6]. However, because kidney transplant recipients have largely been excluded from these studies, robust evidence on the efficacy and safety of these agents in this population remains lacking. Nevertheless, encouraging findings from CKD studies, preclinical studies, small clinical studies, and case reports suggest that these agents may also be beneficial in kidney transplant recipients [7,8,9,10,11,12].

It is well established that kidney transplant recipients have a higher risk of cardiovascular mortality due to multiple factors [13]. Large-scale trials, including EMPA-REG OUTCOME, DAPA-CKD, and EMPA-KIDNEY, have shown that SGLT2i reduce cardiovascular mortality and slow CKD progression in both diabetic and non-diabetic populations. However, transplant recipients were excluded from these trials, leaving limited data for this group.

Data on the long-term effects of SGLT2is in non-diabetic kidney transplant recipients remain limited. Therefore, this study aimed to investigate the effects of SGLT2is on proteinuria and renal function parameters during a 12-month follow-up period in this patient population.

## 2. Materials and Methods

### 2.1. Participants

A database of 1750 kidney transplant recipients from two centers was retrospectively reviewed. Patients were divided into two groups based on SGLT2i use. In routine clinical practice at our centers, SGLT2is were primarily prescribed to kidney transplant recipients with persistent proteinuria and/or chronic allograft dysfunction despite ongoing ACEi or ARB therapy.

### 2.2. Inclusion Criteria

Age between 18 and 75 years;

Kidney transplantation performed at least 1 year prior;

Estimated glomerular filtration rate (eGFR) > 20 mL/min/1.73 m^2^;

Ongoing treatment with an ACEi or ARB therapy;

eGFR < 90 mL/min/1.73 m^2^ or protein–creatinine ratio > 150 mg/g;

Regular use of dapagliflozin or empagliflozin (10 mg) for at least 1 year.

### 2.3. Exclusion Criteria

Rejection episode within the last 3 months;

Surgical intervention within the last 3 months;

Change in immunosuppressive therapy within the last 3 months;

Albumin infusion;

Presence of an active infection.

### 2.4. Study Design

Data from 1750 kidney transplant recipients followed at two centers were retrospectively reviewed. Among this cohort, 25 non-diabetic patients using SGLT2is were identified. Five patients with irregular follow-up and two patients with active infection were excluded. Thus, 18 patients using SGLT2i comprised the study group (Group 1). Among the patients receiving SGLT2i, 14 were treated with empagliflozin and 4 with dapagliflozin, according to routine clinical practice and physician preference. Given the limited sample size, the study was not designed to compare individual SGLT2is. Therefore, all patients receiving SGLT2is were analyzed as a single treatment group to evaluate the overall class effect of SGLT2 inhibition. From the same cohort, 30 kidney transplant recipients who were not using SGLT2is and had similar demographic and biochemical characteristics were selected as the control group (Group 2). Controls were selected based on eligibility criteria without one-to-one matching. To minimize selection bias, the investigators responsible for data collection selected control patients in a blinded manner, without access to patient identities. Control selection was based on achieving the closest possible similarity between groups with respect to baseline demographic characteristics, renal function parameters, proteinuria levels, and other relevant laboratory variables.

A post hoc power analysis was performed using G*Power version 3.1.9.7 based on the comparison of proteinuria change from baseline to 6 months between the two groups. The significance level was set at α = 0.05. With 18 patients in the SGLT2i group and 30 patients in the control group, and using the observed effect size (Cohen’s d ≈ 0.62), the achieved statistical power was approximately 54%. Therefore, the study should be considered exploratory and hypothesis-generating.

**Figure 1:**
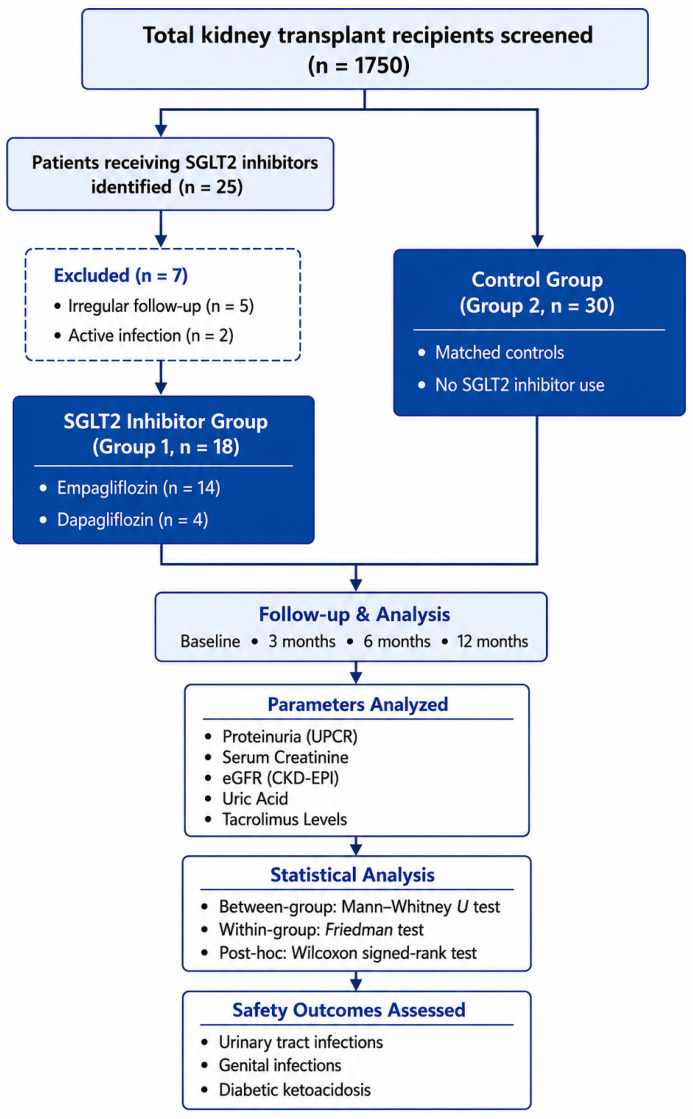
Flowchart of patient selection and study design.

Patients were systematically evaluated for SGLT2i-related complications, including genital infections, leukocyturia, urinary tract infections requiring hospitalization, and ketoacidosis (Figure 1).

### 2.5. Data Collection and Follow-Up

Baseline demographic and clinical data were retrospectively extracted from electronic medical records. Baseline variables included transplant type, serum urea, creatinine, potassium, estimated glomerular filtration rate (eGFR), uric acid, alanine aminotransferase (ALT), C-reactive protein (CRP), and medications. For Group 1, the measurement closest to the time of SGLT2i initiation was considered the baseline value. For Group 2, clinically stable patients with comparable proteinuria, eGFR, and demographic characteristics were selected. Follow-up data from the visits closest to the 3-, 6-, and 12-month time points were also analyzed. Eligible patients identified during the 10 years preceding ethics committee approval were included in the analysis. Patient data were analyzed only after obtaining ethics committee approval. The requirement for informed consent was waived by the ethics committee because of the retrospective, non-interventional nature of the study.

eGFR was calculated using the CKD-EPI formula. Proteinuria was assessed by the urine protein-to-creatinine ratio (UPCR) measured in morning spot urine samples collected during routine follow-up visits.

Blood pressure measurements were recorded; however, due to heterogeneity in measurement methods (home versus clinic measurements and manual versus automated devices), these data were excluded from the final analyses.

### 2.6. Ethical Approval

The study was approved by the local ethics committee on 28 January 2026 (approval number: 2024-TBEK 2026/01-06). The study was conducted in accordance with the Declaration of Helsinki and Good Clinical Practice guidelines.

### 2.7. Statistical Analysis

Normality of continuous variables was assessed using the Shapiro–Wilk test. Non-parametric tests were used for non-normally distributed variables. Descriptive statistics were presented as percentages for categorical variables and as median (min–max) for continuous variables.

Between-group comparisons were performed using the Mann–Whitney U test. The Friedman test was used for repeated measures within groups, followed by the Wilcoxon signed-rank test for post hoc analyses when appropriate. Categorical variables were compared using the chi-square test.

All analyses were performed using IBM SPSS Statistics version 26.0 (IBM Corp., Armonk, NY, USA). A *p*-value < 0.05 was considered statistically significant.

## 3. Results

No significant differences were observed between the groups in terms of age, sex, smoking status, body weight, or CKD etiology. Baseline biochemical parameters, including BUN, creatinine, eGFR, uric acid, potassium, ALT, and tacrolimus levels, were also comparable between groups (*p* > 0.05) (Table 1).

**Figure 2:**
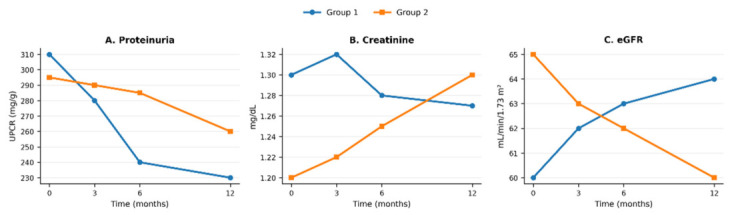
Changes in proteinuria (**A**), serum creatinine (**B**), and eGFR (**C**) over time in the study groups. Median values are presented at baseline, 3, 6, and 12 months. Group 1 (SGLT2 inhibitor group) is represented by the blue line and Group 2 (control group) by the orange line.

In Group 1, proteinuria decreased by approximately 20% at 6 months and 26% at 12 months compared with baseline. However, these reductions did not reach statistical significance within the group. The reduction in proteinuria from baseline to 6 months was significantly greater in Group 1 than in Group 2 (*p* = 0.037). Although the downward trend persisted at 12 months, the difference between groups was no longer statistically significant (*p* > 0.05) (Table 2, Figure 2).

In Group 2, proteinuria decreased by approximately 3% at 6 months and 10% at 12 months; however, these changes were not statistically significant (*p* > 0.05). No significant changes in serum creatinine or eGFR were observed in either group during the 12-month follow-up. Likewise, no significant between-group differences were observed in these parameters (Table 2, Figure 2).

Tacrolimus levels remained stable during follow-up, with no clinically significant differences between groups (*p* > 0.05).

Uric acid levels were significantly lower in Group 1 than in Group 2 at month 3 (*p* = 0.031). In Group 2, no significant change in uric acid levels was observed throughout the follow-up period. At month 12, no significant difference between groups was observed.

During follow-up, no infections requiring hospitalization or cases of diabetic ketoacidosis were observed. Two patients in Group 1 developed uncomplicated urinary tract infections and were treated on an outpatient basis. No significant differences in infection-related adverse events were observed between groups.

## 4. Discussion

In this exploratory and hypothesis-generating study of non-diabetic kidney transplant recipients with CKD, SGLT2i use was associated with a trend toward reduced proteinuria without apparent adverse effects on renal function or safety outcomes. Notably, the reduction in proteinuria from baseline to 6 months was significantly greater in the SGLT2i group than in the control group.

Proteinuria is one of the strongest predictors of graft damage and long-term graft loss in kidney transplant recipients, as in other forms of CKD [14,15,16]. Observational and prospective studies have shown that post-transplant proteinuria is associated with poorer patient and graft survival [17,18]. Consequently, reducing proteinuria remains a major therapeutic goal in kidney transplant recipients.

The renoprotective effects of SGLT2is have been well established in large randomized trials, including CREDENCE, DAPA-CKD, and EMPA-KIDNEY. These studies reported reductions in proteinuria of approximately 20–35% and improved long-term renal outcomes [17,18,19]. However, kidney transplant recipients were excluded from these trials, leaving limited evidence for this population. Available studies in kidney transplant recipients have generally been small, observational, and restricted to diabetic patients [13,20,21,22]. Although these studies have suggested reductions in proteinuria together with improvements in glycemic and metabolic parameters, data in non-diabetic transplant recipients remain scarce.

In our study, proteinuria decreased by approximately 20% at 6 months and 26% at 12 months in the SGLT2i group. These findings are consistent with the antiproteinuric effects reported in large CKD trials and suggest that SGLT2i may have similar effects in non-diabetic kidney transplant recipients. The significantly greater reduction in proteinuria observed at 6 months compared with the control group may indicate an early antiproteinuric effect in this population. However, the between-group difference was no longer statistically significant at 12 months. Given the limited sample size and retrospective design, these findings should be interpreted with caution and require confirmation in larger prospective studies.

In patients with diabetes, proteinuria is often associated with mesangial expansion, glomerular basement membrane thickening, and progressive structural kidney damage. By contrast, in non-diabetic kidney transplant recipients, proteinuria is thought to be driven predominantly by hemodynamic factors, including hyperfiltration, increased intraglomerular pressure, and calcineurin-inhibitor-related effects [23,24]. Consequently, these patients may be particularly responsive to hemodynamically active agents such as SGLT2is. Mechanistically, SGLT2is reduce sodium and glucose reabsorption in the proximal tubule, thereby increasing distal sodium delivery. This enhances tubuloglomerular feedback and reduces intraglomerular pressure [24,25]. In addition, experimental and clinical studies have suggested anti-inflammatory, antifibrotic, and antioxidative effects that may contribute to renal protection [23,26,27,28]. Other proposed mechanisms include improvements in mitochondrial function, increased erythropoietin production, and reduced renal hypoxia [23,24,25,26]. Although these mechanisms are likely to operate in kidney transplant recipients, their effects may be modified by immunosuppressive therapy, calcineurin inhibitor exposure, and pathophysiological processes specific to chronic allograft injury. Nevertheless, the antiproteinuric effect observed in our non-diabetic cohort is consistent with the concept that the renal benefits of SGLT2is are not solely dependent on glycemic control.

The effects of SGLT2is on renal function are also of particular interest in kidney transplant recipients. No significant decline in eGFR was observed during follow-up among patients receiving SGLT2is. In the general CKD population, SGLT2is are known to cause an initial transient decline in eGFR, followed by long-term preservation of renal function [17,18,19,23]. In the DAPA-CKD and EMPA-KIDNEY trials, these agents maintained their efficacy and safety even in patients with low eGFR levels [23,24,25]. In our study, stable eGFR and serum creatinine levels, together with the favorable proteinuria profile, provide preliminary reassurance regarding the renal safety of SGLT2is in non-diabetic kidney transplant recipients.

Data regarding the effects of SGLT2is on calcineurin inhibitor levels in transplant recipients remain limited. Observational studies in diabetic kidney transplant recipients have shown stable tacrolimus levels following initiation of SGLT2i therapy. Consistent with these findings, no significant change in tacrolimus levels was observed during the 12-month follow-up in our study, suggesting that pharmacokinetic interactions between SGLT2is and calcineurin inhibitors may be limited. The potential interaction between SGLT2is and calcineurin inhibitors may extend beyond pharmacokinetic effects. Calcineurin inhibitors are known to contribute to nephrotoxicity by increasing oxidative stress, activating TGF-β signaling, and promoting tubulointerstitial fibrosis [29,30]. Experimental studies have shown that empagliflozin and dapagliflozin may attenuate tacrolimus-induced nephrotoxicity by suppressing TGF-β/Smad pathways and reducing inflammation and oxidative stress [27,28]. These observations suggest a potential renoprotective role of SGLT2i in transplant recipients receiving calcineurin inhibitors.

In addition to their effects on proteinuria and renal function, SGLT2is have been associated with several metabolic benefits, including reductions in serum uric acid levels. In our study, the lower uric acid levels observed at month 3 in the SGLT2i group are consistent with the known uricosuric effects of these agents; however, this difference was not maintained at month 12.

The most important adverse effects associated with SGLT2is are urinary tract infections, genital infections, and diabetic ketoacidosis. Although the risk of infection may theoretically be concerning in kidney transplant recipients receiving immunosuppressive therapy, the limited available literature suggests that these agents have an acceptable safety profile in this population [20,21,22,31]. In our study, no severe urinary tract infections, genital infections, or cases of diabetic ketoacidosis were observed among non-diabetic patients receiving SGLT2i. Two patients developed uncomplicated urinary tract infections, and no significant difference in infection frequency was found between groups. No major safety concerns were identified during the 12-month follow-up period.

Despite these encouraging findings, larger prospective studies are needed to better define the efficacy and safety of SGLT2is in kidney transplant recipients. The INFINTI trial (ClinicalTrials.gov Identifier: NCT04965935) and the LIFECYCLE trial (ClinicalTrials.gov Identifier: NCT05374291), both evaluating dapagliflozin in kidney transplant recipients, are currently underway. The results of these studies are expected to provide more robust evidence regarding the renal and metabolic effects of SGLT2is in this population.

The main limitations of our study include its retrospective design, the exclusion of blood pressure measurements from the analysis, and the relatively small sample size. Although blood pressure is an important determinant of both proteinuria and graft outcomes, measurements were retrospectively obtained from routine clinical practice and were not standardized across centers or study visits. Variability in measurement settings, devices, and recording methods was considered likely to affect data reliability; therefore, these data were not included in the formal statistical analyses.

In addition, the possibility of confounding by indication should be acknowledged, as SGLT2is were preferentially prescribed to patients with persistent proteinuria and/or chronic allograft dysfunction in routine clinical practice. Because of the limited sample size, the study may have been underpowered to detect differences in some outcomes. Accordingly, the findings should be interpreted with caution and considered hypothesis-generating rather than definitive.

## 5. Conclusions

In conclusion, our findings suggest that SGLT2is may represent a feasible and well-tolerated therapeutic option for selected non-diabetic kidney transplant recipients. However, larger prospective studies are needed to confirm their long-term safety and efficacy.

## Figures and Tables

**Table 1:** Baseline demographic and biochemical characteristics.

Variables	Group 1 (*n* = 18)	Group 2 (*n* = 30)	*p* *
Age (years)	53.5 (35–71)	52.0 (28–73)	>0.05
Female sex, *n*	15	24	>0.05
Smoking status, *n*	0	2	>0.05
BUN (mg/dL)	21 (8–152)	22 (10–65)	>0.05
Serum creatinine (mg/dL)	1.3 (0.6–3.26)	1.2 (0.68–3.0)	>0.05
eGFR (mL/min/1.73 m^2^)	60 (20–110)	65 (20–105)	>0.05
Proteinuria (mg/g)	310 (120–980)	295 (110–1020)	>0.05
Tacrolimus (ng/mL)	6.5 (3–12)	6.8 (2.5–13)	>0.05
ACEI/ARB use (%)	100	100	>0.05
Hypertension *n* (%)	16 (88.9)	21 (70.0)	>0.05
Stone disease, *n* (%)	3 (16.7)	4 (13.3)	>0.05
Living donor transplantation, *n* (%)	6 (33%)	10 (33%)	>0.05

Note: Values are presented as median (minimum–maximum). * *p*-values represent comparisons between groups. Mann–Whitney U test was used for continuous variables and Fisher’s exact test for categorical variables. (Group 1 vs. Group 2). A *p*-value < 0.05 was considered statistically significant. eGFR: estimated glomerular filtration rate; BUN: blood urea nitrogen.

**Table 2:** Longitudinal changes in biochemical parameters according to study groups.

Parameter—Time Point	Group 1 (*n* = 18)	Group 2 (*n* = 30)	*p* *
**Proteinuria (mg/g)**			
Baseline	310 (120–980)	295 (110–1020)	0.41
Month 3	280 (100–850)	290 (120–990)	0.52
Month 6	240 (90–780)	285 (115–970)	0.33
Month 12	230 (85–750)	260 (110–940)	0.28
**Δ Proteinuria**			
0–3	−20 (−150–60)	−5 (−120–90)	0.18
**0–6**	**−60 (−300–40)**	**−10 (−180–120)**	**0.037**
0–12	−80 (−400–30)	−30 (−250–150)	0.11
**Serum creatinine (mg/dL)**			
Baseline	1.30 (0.60–3.26)	1.20 (0.70–3.00)	0.62
Month 3	1.32 (0.65–3.10)	1.22 (0.70–2.90)	0.59
Month 6	1.28 (0.60–3.00)	1.25 (0.75–3.10)	0.71
Month 12	1.27 (0.58–2.95)	1.30 (0.80–3.20)	0.54
**eGFR (mL/min/1.73 m^2^)**			
Baseline	60 (20–110)	65 (20–105)	0.47
Month 3	62 (20–108)	63 (22–102)	0.61
Month 6	63 (22–105)	62 (20–100)	0.74
Month 12	64 (25–102)	60 (20–98)	0.38
**Tacrolimus level (ng/mL)**			
Baseline	6.5 (3–12)	6.8 (2.5–13)	0.75
Month 3	6.2 (3–11)	6.5 (2.8–12)	0.68
Month 6	6.0 (2.5–10.5)	6.3 (3–11.5)	0.56
Month 12	5.8 (2–10)	6.2 (2.5–11)	0.49
**Uric acid (mg/dL)**			
Baseline	6.8 (4–10)	7.2 (4.5–11)	0.23
**Month 3**	**6.0 (3.5–9)**	**7.0 (4–10.5)**	**0.031**
Month 6	6.2 (3.8–9.5)	6.8 (4.2–10)	0.12
Month 12	6.5 (4–9.8)	6.7 (4.5–10.2)	0.44

Note: Values are presented as median (minimum–maximum). * *p*-values were calculated using the Mann–Whitney U test for between-group comparisons (Group 1 vs. Group 2). Changes over time within groups were analyzed using the Friedman test. eGFR: estimated glomerular filtration rate (calculated using the CKD-EPI equation). A *p*-value < 0.05 was considered statistically significant. Bold values indicate statistical significance (*p* < 0.05).

## Data Availability

The data that support the findings of this study are available from the corresponding author upon reasonable request.

## Abbreviations

ACEi	Angiotensin-converting enzyme inhibitor
ARB	Angiotensin receptor blocker
ALT	Alanine aminotransferase
BUN	Blood urea nitrogen
CKD	Chronic kidney disease
CRP	C-reactive protein
eGFR	Estimated glomerular filtration rate
GLP-1	Glucagon-like peptide-1
MRA	Mineralocorticoid receptor antagonist
SGLT2i	Sodium–glucose cotransporter 2 inhibitor

## References

[1] Chaudhry D., Chaudhry A., Peracha J., Sharif A. (2022). Survival for waitlisted kidney failure patients receiving transplantation versus remaining on waiting list: Systematic review and meta-analysis. BMJ.

[2] Minkovich M., Gupta N., Liu M., Famure O., Li Y., Selzner M., Lee J.Y., Kim S.J., Ghanekar A. (2024). Impact of early surgical complications on kidney transplant outcomes. BMC Surg..

[3] American Diabetes Association Professional Practice Committee (2024). Chronic kidney disease and risk management: Standards of care in diabetes—2024. Diabetes Care.

[4] de Boer I.H., Khunti K., Sadusky T., Tuttle K.R., Neumiller J.J., Rhee C.M., Rosas S.E., Rossing P., Bakris G. (2022). Diabetes management in chronic kidney disease: A consensus report by the American Diabetes Association (ADA) and Kidney Disease: Improving Global Outcomes (KDIGO). Diabetes Care.

[5] Pitt B., Filippatos G., Agarwal R., Anker S.D., Bakris G.L., Rossing P., Joseph A., Kolkhof P., Nowack C., Schloemer P. (2021). Cardiovascular events with finerenone in kidney disease and type 2 diabetes. N. Engl. J. Med..

[6] Bakris G.L., Agarwal R., Anker S.D., Pitt B., Ruilope L.M., Rossing P., Kolkhof P., Nowack C., Schloemer P., Joseph A. (2020). Effect of finerenone on chronic kidney disease outcomes in type 2 diabetes. N. Engl. J. Med..

[7] Abdelhakim A.M., Abd-ElGawad M. (2020). Impact of mineralocorticoid receptor antagonists in renal transplant patients: A systematic review and meta-analysis. J. Nephrol..

[8] de Sousa M.V., Guida J.P., do Valle C.F., Camargo L.F., Rivelli G.G., Mazzali M. (2017). Spironolactone in post-transplant proteinuria: A safe alternative therapy. Transplant. Proc..

[9] Baskin E., Siddiqui M.A., Gülleroğlu K., Özdemir B.H., Yılmaz A.Ç., Çolak M.Y., Akdur A., Soy E.A., Moray G., Haberal M. (2023). Long-term effect of eplerenone treatment in children with chronic allograft nephropathy. Pediatr. Transplant..

[10] Waanders F., Rienstra H., Boer M.W., Zandvoort A., Rozing J., Navis G., van Goor H., Hillebrands J.L. (2009). Spironolactone ameliorates transplant vasculopathy in renal chronic transplant dysfunction in rats. Am. J. Physiol. Ren. Physiol..

[11] Afsar B., Afsar R.E., Caliskan Y., Lentine K.L. (2025). Mineralocorticoid receptor blockage in kidney transplantation: Too much of a good thing or not?. Int. Urol. Nephrol..

[12] Kahvecioglu S., Celik H., Yalcinkaya A.S., Ayar Y., Aktas N., Akarsu O. (2025). Efficacy and safety of finerenone in kidney transplant patients. J. Clin. Med..

[13] Boonpiraks K., Krisanapan P., Anumas S. (2023). Efficacy and safety of sodium-glucose cotransporter-2 inhibitors in diabetic kidney transplant recipients: A systematic review and meta-analysis. Clin. Transplant..

[14] Halimi J.M., Laouad I., Buchler M., Al-Najjar A., Chatelet V., Houssaini T.S., Nivet H., Lebranchu Y. (2005). Early low-grade proteinuria: Causes, short-term evolution and long-term consequences in renal transplantation. Kidney Int..

[15] Amer H., Fidler M.E., Myslak M., Morales P., Kremers W.K., Larson T.S., Stegall M.D., Cosio F.G. (2007). Proteinuria after kidney transplantation: Relationship to allograft histology and survival. J. Am. Soc. Nephrol..

[16] Opelz G., Döhler B. (2006). Association between steroid dosage and graft survival in kidney transplantation. Transplantation.

[17] Perkovic V., Jardine M.J., Neal B., Bompoint S., Heerspink H.J., Charytan D.M., Edwards R., Agarwal R., Bakris G., Bull S. (2019). Canagliflozin and renal outcomes in type 2 diabetes and nephropathy. N. Engl. J. Med..

[18] Heerspink H.J., Stefánsson B.V., Correa-Rotter R., Chertow G.M., Greene T., Hou F.F., Mann J.F., McMurray J.J., Lindberg M., Rossing P. (2020). Dapagliflozin in patients with chronic kidney disease. N. Engl. J. Med..

[19] Herrington W.G., Staplin N., Wanner C., Green J.B., Hauske S.J., Emberson J.R., Preiss D., Judge P., Mayne K.J., Ng S.Y.A. (2023). Empagliflozin in patients with chronic kidney disease. N. Engl. J. Med..

[20] Halden T.A., Kvitne K.E., Midtvedt K., Rajakumar L., Robertsen I., Brox J., Bollerslev J., Hartmann A., Åsberg A., Jenssen T. (2019). Efficacy and safety of empagliflozin in renal transplant recipients with post-transplant diabetes mellitus. Diabetes Care.

[21] Schwaiger E., Burghart L., Signorini L., Ristl R., Kopecky C., Tura A., Pacini G., Wrba T., Antlanger M., Schmaldienst S. (2019). Empagliflozin in post-transplant diabetes mellitus: A prospective observational pilot study. Am. J. Transplant..

[22] Lim J.H., Kwon S., Jeon Y., Kim H.Y., Kwon H., Kim Y.S., Lee H., Kim Y.L., Kim C.D., Park S.H. (2022). The Efficacy and Safety of SGLT2 Inhibitor in Diabetic Kidney Transplant Recipients. Transplantation.

[23] Heerspink H.J., Perkins B.A., Fitchett D.H., Husain M., Cherney D.Z. (2016). Sodium-glucose cotransporter 2 inhibitors in the treatment of diabetes mellitus: Cardiovascular and kidney effects. Circulation.

[24] Vallon V., Thomson S.C. (2017). Targeting renal glucose reabsorption to treat hyperglycaemia: The pleiotropic effects of SGLT2 inhibition. Diabetes.

[25] Zelniker T.A., Braunwald E. (2020). Mechanisms of cardiorenal effects of sodium-glucose cotransporter 2 inhibitors. Circulation.

[26] Dekkers C.C., Petrykiv S., Laverman G.D., Cherney D.Z., Gansevoort R.T., Heerspink H.J. (2018). Effects of the SGLT2 inhibitor dapagliflozin on glomerular and tubular injury markers. Kidney Int..

[27] Jin J., Jin L., Luo K., Lim S.W., Chung B.H., Yang C.W. (2017). Effect of Empagliflozin on Tacrolimus-Induced Pancreas Islet Dysfunction and Renal Injury. Am. J. Transplant..

[28] Ko E.J., Shin Y.J., Cui S., Lim S.W., Chung B.H., Yang C.W. (2022). Effect of dual inhibition of DPP4 and SGLT2 on tacrolimus-induced diabetes mellitus and nephrotoxicity in a rat model. Am. J. Transplant..

[29] Farouk S.S., Rein J.L. (2020). The Many Faces of Calcineurin Inhibitor Toxicity—What the FK?. Adv. Chronic Kidney Dis..

[30] Eberhardt W., Doller A., Pfeilschifter J. (2018). Calcineurin inhibitors and renal fibrosis: Mechanisms and therapeutic perspectives. Cell Signal.

[31] Li D., Wang T., Shen S., Fang Z., Dong Y., Tang H. (2017). Urinary tract and genital infections in patients with type 2 diabetes treated with sodium-glucose cotransporter 2 inhibitors: A meta-analysis of randomized controlled trials. Diabetes Obes. Metab..

